# The Mental Health of Elite Athletes: A Narrative Systematic Review

**DOI:** 10.1007/s40279-016-0492-2

**Published:** 2016-02-20

**Authors:** Simon M. Rice, Rosemary Purcell, Stefanie De Silva, Daveena Mawren, Patrick D. McGorry, Alexandra G. Parker

**Affiliations:** 1 Orygen, The National Centre of Excellence in Youth Mental Health, 35 Poplar Road, Parkville, Melbourne, VIC 3052 Australia; 2 Centre for Youth Mental Health, The University of Melbourne, Melbourne, VIC Australia; 3 Orygen Youth Health Clinical Program, Melbourne, VIC Australia

## Abstract

**Background:**

The physical impacts of elite sport participation have been well documented; however, there is comparatively less research on the mental health and psychological wellbeing of elite athletes.

**Objective:**

This review appraises the evidence base regarding the mental health and wellbeing of elite-level athletes, including the incidence and/or nature of mental ill-health and substance use.

**Methods:**

A systematic search of the PubMed, EMBASE, SPORTDiscus, PsycINFO, Cochrane and Google Scholar databases, up to and including May 2015, was conducted.

**Results:**

The search yielded a total of 2279 records. Following double screening, 60 studies were included. The findings suggested that elite athletes experience a broadly comparable risk of high-prevalence mental disorders (i.e. anxiety, depression) relative to the general population. Evidence regarding other mental health domains (i.e. eating disorders, substance use, stress and coping) is less consistent. These results are prefaced, however, by the outcome of the quality assessment of the included studies, which demonstrated that relatively few studies (25 %) were well reported or methodologically rigorous. Furthermore, there is a lack of intervention-based research on this topic.

**Conclusion:**

The evidence base regarding the mental health and wellbeing of elite athletes is limited by a paucity of high-quality, systematic studies. Nonetheless, the research demonstrates that this population is vulnerable to a range of mental health problems (including substance misuse), which may be related to both sporting factors (e.g. injury, overtraining and burnout) and non-sporting factors. More high-quality epidemiological and intervention studies are needed to inform optimal strategies to identify and respond to player mental health needs.

**Electronic supplementary material:**

The online version of this article (doi:10.1007/s40279-016-0492-2) contains supplementary material, which is available to authorized users.

## Key Points

**Table:** 

The evidence base regarding the mental health and wellbeing of elite athletes is limited by a paucity of high-quality, systematic studies, including intervention trials.
On the basis of current evidence, elite athletes appear to experience a broadly comparable risk of high-prevalence mental disorders relative to the general population. A greater risk of disorder may be experienced by elite athletes who are injured, approaching/in retirement or experiencing performance difficulty.
While the importance of elite athlete mental health is gaining increasing attention, targeted, disorder-specific models of care are yet to be established for this group. There is scope for such models to capitalise on early-intervention principles and establish cross-discipline collaboration.

## Introduction

High-quality, systematic studies on the nature and impacts of physical injuries in elite athletes—most notably, head injuries/concussion and limb injuries—have led to advances in how these injuries are optimally managed or, ideally, prevented. There is comparatively less research on, but growing interest in, the mental health and psychological wellbeing of elite-level athletes [1–3]. The prevalence of diagnosable psychiatric disorders in this population remains a matter of debate [4]; however, notions that elite athletes are devoid of mental health problems have been increasingly scrutinised by sports medicine practitioners [5].

The intense mental and physical demands placed on elite athletes are a unique aspect of a sporting career, and these may increase their susceptibility to certain mental health problems and risk-taking behaviours [9]. Furthermore, the peak competitive years for elite athletes [10] tend to overlap with the peak age for the risk of onset of mental disorders [11, 12]. In addition to physical and competition stress, elite athletes face a unique array of ‘workplace’ stressors, including the pressures of increased public scrutiny through mainstream and social media, limited support networks due to relocation, group dynamics in team sports and the potential for injuries to end careers prematurely [13–17]. The ways by which athletes appraise and cope with these stressors can be a powerful determinant of the impact the stressors have on both their mental health and their sporting success [18].

Athletes tend not to seek support for mental health problems, for reasons such as stigma, lack of understanding about mental health and its potential influence on performance, and the perception of help seeking as a sign of weakness [12, 19]. While there have been efforts to disseminate sport-related mental health findings in order to advance the prevention, identification and early treatment of psychopathology in elite athletes, there are suggestions that some sporting governing bodies continue to minimise the significance of mental ill-health in this population [19]. This has sobering implications if elite athletes within such organisations are not provided with access to timely or adequate mental health care, or do not feel that the culture of the sporting organisation is such that they can even raise their mental health concerns. While it is well established that physical activity has a positive effect on mental health [6, 7], a review has found that intense physical activity performed at the elite athlete level might instead *compromise* mental wellbeing, increasing symptoms of anxiety and depression through overtraining, injury and burnout [8]. Some, though not all, research suggests that this population has an increased risk of mental health problems, including eating disorders [21] and suicide [22]. A recent national survey of elite athletes in Australia found that almost half acknowledged symptoms of at least one of the mental health problems that were assessed, with prevalence rates similar to those reported in the community [23]. Emerging research suggests that retired elite athletes may be at particularly elevated risk of mental ill-health [24], corresponding to both low rates of formal athlete mental health screening processes [25] and player perceptions of inadequate availability of mental health support [26].

Given the early-stage state of sports psychiatry and its research base, the current delivery of mental health care for elite athletes might not take into account sport-related factors that potentially influence vulnerability to mental health problems, nor diagnostic or treatment issues that may be unique to this population [4, 19]. Developing a comprehensive understanding of the mental health and psychological wellbeing specific to elite athletes has the potential to advance models of care and management of this population, which may, in turn, facilitate performance gains. Such an understanding is required to provide guidance for sport practitioners—including coaches, medical staff and sport psychologists—in developing the coping abilities of elite athletes and, in turn, improving their emotional wellbeing [20].

### Objective

The utility of systematic reviews to synthesise research on discrete topics and identify gaps in knowledge is well established; however, to date, there have been no such reviews of the mental health and psychological wellbeing of elite athletes. The objective of this review was to synthesise the growing evidence base regarding the incidence and nature of mental ill-health (including substance use) and psychological wellbeing among elite-level athletes in order to identify gaps that future research should prioritise, and inform strategies or guidelines to advance the detection and management of mental ill-health in this population.

## Methods

### Literature Search

A systematic search of five electronic databases (PubMed, EMBASE, SPORTDiscus, PsycINFO, Cochrane) was conducted between January and February 2015, using the relevant database search engines. A subsequent search was conducted using the Google Scholar database in May 2015 to ensure that all recently published articles meeting the inclusion criteria were identified. The search strategy for each database, MeSH descriptors and corresponding number of hits per database are presented in Electronic Supplementary Material Tables S1 and S2.

### Study Inclusion

**Fig. 1:**
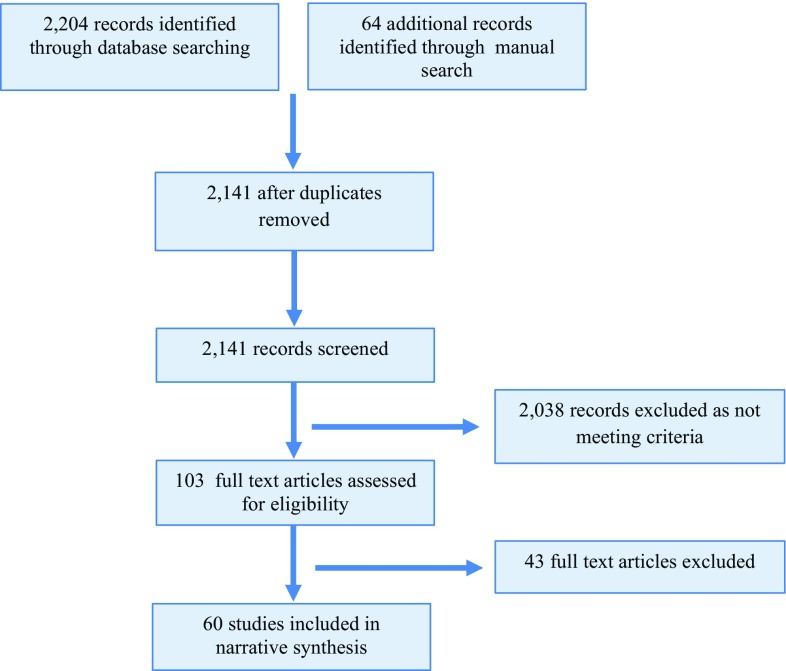
Study selection flow diagram

Three researchers independently assessed the eligibility of each retrieved record on the basis of the title and abstract. If the information was unclear, the full-text article was screened. All included studies were subsequently re-screened (i.e. double screened) by a fourth researcher. The included studies were required to meet the following inclusion criteria: (1) participants were currently competing at the elite level, as able-bodied athletes, where the elite level was defined a priori to be competitive at either the Olympic, international, national or professional level; (2) the study reported quantitative data on a mental health, wellbeing or coping outcome; and (3) the study was published in English. Studies were excluded from the review on the basis of the following criteria: (1) the mean age of the participants was <18 years; (2) participants were competing at a school or collegiate level; (3) the study was undertaken with a heterogeneous sample (i.e. a mixed sample of elite and non-elite athletes) without reporting group findings separately; (4) the study assessed only physiological wellbeing or stress responses without assessing or reporting psychological wellbeing; (5) the study was available in abstract form only (i.e. conference presentations), precluding full quality assessment; and (6) the study described substance use focused on performance enhancement (i.e. doping) as opposed to personal use. The systematic review was conducted in accordance with the Preferred Reporting Items for Systematic Reviews and Meta-Analyses (PRISMA) guidelines (see Fig. 1 for flow diagram).

### Data Extraction

A standardised data extraction template was designed. One researcher sourced the required information from the included studies, using this template, including the study type and design, sport population, study aim, sex ratio and key outcomes mapped to study measures and main findings.

### Quality Appraisal

Given the heterogeneity of the included study designs and the lack of randomised, controlled trials identified in the search process (see Sect. 3.2), it was not possible to conduct a standard risk-of-bias assessment. In place of this, all studies were appraised for reporting quality based on the established standards outlined below.

## Results

### Literature Search

The literature search yielded a total of 2279 records. After screening of the titles and abstracts, 103 studies were identified as likely meeting the inclusion criteria. After double screening, exclusion of qualitative studies and a final manual search of the literature (i.e. screening of references lists), a total of 60 studies were included in the quantitative synthesis (see Fig. 1 for the study selection diagram).

### Study Design

Sixty quantitative studies were included in the review. The study designs varied, though most were either cross-sectional observational studies (*N* = 38; 63.3 %), longitudinal studies (*N* = 11; 18.3 %) or of a mixed-method design (*N* = 8; 13.3 %). In addition, there were one randomised, controlled trial (1.6 %), one meta-analysis and one intervention case study report. Given the heterogeneity of both the study designs and the outcome variables that were assessed, it was not possible to conduct a meta-analysis as part of this review.

### Quality Appraisal

The methodological rigour of the included studies was assessed according to relevant published criteria [27, 28]. Quality appraisal of the 60 studies is presented in Electronic Supplementary Material Table S3. Only two studies met all methodological criteria, with one quarter (*N* = 15; 25 %) assessed to be of good reporting quality (i.e. scoring ≥4 out of 5). The mean quality rating was 2.88 (standard deviation 0.87). Over one third of the remaining studies (*N* = 21; 45 %) were assessed to be of moderate quality (scoring 3 out of 5), while 24 studies (40 %) were assessed to be of low quality (scoring 2 out of 5). Almost all included studies (*N* = 59) defined their participants well and either reported use of standardised, validated questionnaires or clearly described the outcomes measured. Fewer than half of the included studies clearly reported ethical review (*N* = 27) and either reported a participant rate of more than 80 % or, in the absence of that, provided a description comparing responders with non-responders (*N* = 25). Over one quarter of the included studies did not report on the participant rate (*N* = 16), and very few used random sampling (*N* = 3).

### Description of Included Studies

Tables 1, 2, 3, 4, 5, 6, 7 provide a summary of the key characteristics and main outcomes of the 60 included studies. For the purposes of reporting and analysis, studies were grouped according to the following major mental health constructs: anger and aggression (*N* = 2) [29, 30], anxiety (*N* = 4) [31–34], eating disorder and body image (*N* = 10) [21, 35–43], general-prevalence studies (*N* = 10) [23, 44–52], help seeking (*N* = 1) [11], sleep (*N* = 1) [53], stress and coping (*N* = 22) [64–86], substance use (*N* = 9) [54–62] and wellbeing (*N* = 1) [63]. The included studies examined athletes from a broad range of individual sports (e.g. swimming, tennis, wrestling) and team-based sports (e.g. soccer, football, rugby), with some studies including elite athletes from a range of sporting disciplines.

**Table 1:** Summary of anger and aggression studies in elite athletes

Authors	Type	Purpose	*N* (male:female)	Sport; country	Main findings
Robazza and Bortoli [29]	Quantitative; cross-sectional, observational	Perceived effects of trait anger	197 (197:0)	Rugby; Italy	Players experienced a moderate frequency of anger symptoms, interpreting these as facilitative rather than debilitative. Anxiety was a significant predictor of anger, while self-confidence was a significant predictor of control of anger. High- and low-level competitors did not differ in their frequency and interpretation of anger symptoms
Si and Lee [30]	Quantitative; case study, intervention (no control)	Mental skills training for anger	1 (1:0)	Table tennis, Hong Kong	Results supported the effectiveness of psychological intervention in changing the athlete’s low frustration tolerance behaviours directed towards others. Problem resolution and disputing and restructuring irrational beliefs facilitated performance enhancement in competitions

**Table 2:** Summary of anxiety studies in elite athletes

Authors	Type	Purpose	*N* (male:female)	Sport; country	Main findings
Abrahamsen et al. [31]	Quantitative; cross-sectional, observational	Achievement motivation for performance anxiety	190 (101:89)	Various; Norway	Females reported greater performance worry, concentration disruption and somatic anxiety than males. Perceptions of a performance climate predicted performance worry for both sexes and concentration disruption for females. Perceived ability predicted less performance worry for females and males
Hatzigeorgiadis and Chroni [32]	Quantitative; longitudinal, observational	Pre-competition anxiety and coping	9 (9:0)	Swimming; Greece	Facilitative perceptions of anxiety symptoms were related to more adaptive cognitive and behavioural outcomes. Swimmers perceiving their anxiety states as facilitative reported more approach- and less avoidance-coping strategies than swimmers perceiving their anxiety states as debilitative
Jones et al. [33]	Quantitative; cross-sectional, observational	Anxiety and performance	211 (sex not reported), 97 elite athletes	Swimming; UK	Elite performers interpreted anxiety as more facilitative to performance than non-elite performers. Furthermore, self-confidence was higher in the elite group. Findings supported the distinction between intensity and direction of competitive state anxiety symptoms
Koivula et al. [34]	Quantitative; cross-sectional, observational	Effects of anxiety, self-confidence, self-esteem	178 (109:69)	Various; Sweden	Self-esteem based on respect for self was associated with more positive patterns of perfectionism, while self-esteem dependent on competence aspects showed more negative perfectionism. Negative patterns of perfectionism were related to higher levels of cognitive anxiety and lower levels of self-confidence

**Table 3:** Summary of eating disorder (ED) and body image studies in elite athletes

Authors	Type	Purpose	*N* (male:female)	Sport; country	Main findings
Byrne and McLean [35]	Quantitative; cross-sectional, observational	EDs, elite athletes and non-athletes	263 (108:155) elite athletes, 263 matched controls	Various; Australia	Results suggested that athletes have a higher prevalence of EDs, especially in sports emphasising thin shape (leanness) or low weight. Rates of EDs were higher in female athletes. Athletes competing in sports that emphasise the importance of a thin body shape or low body weight appear to be particularly vulnerable
Filaire et al. [36]	Quantitative; cross-sectional, observational	Maintenance of body weight and risk of EDs	44 (44:0) [27 elite athletes, 17 controls]	Judo and cycling; France	4 % of athletes reported self-induced vomiting, 10 % reported use of laxatives and 8.5 % reported use of diet pills. Athletes reported greater negative feelings about their physical appearance and their body weight satisfaction than controls (*P* < 0.01 and *P* < 0.05, respectively). Depression accounted for >60 % of bulimia and ED scores
Hausenblas and Symons Downs [37]	Quantitative; meta-analysis	Literature review; body image in athletes and non-athletes	19 studies with elite athletes, sex not reported	Various; various	Overall findings highlighted that athletes had a more positive body image than non-athletes. The effect size for this difference was small. Overall, there was no difference between male athletes and female athletes or according to age or body mass index
Hulley and Hill [38]	Quantitative; cross-sectional, observational	EDs and general health and wellbeing	181 (0:181)	Distance runners; UK	Levels of anorexia nervosa and EDNOS were higher than expected in similarly aged, non-athletic women. 29 athletes (16 %) had an ED at the time of the study, 6 had received previous treatment for an ED. The demands for leanness rather than exercise intensity appeared to be the main risk in these elite runners
Jonnalagadda et al. [39]	Quantitative; cross-sectional, observational	Food preferences, body image perceptions, dieting	49 (23:26)	Figure skating; USA	Disordered eating and preference for a thin body contour were reported among this group of athletes, particularly in females. 44 % of males considered themselves overweight. Of the females, 30 % considered themselves overweight and 77 % were terrified about gaining weight. Females exhibited a higher body dissatisfaction score than their male counterparts
Sundgot-Borgen [40]	Quantitative; cross-sectional, observational	Risk factors for EDs in female athletes	603 (0:603)	Various; Norway	Rate of EDs was higher in sports emphasising leanness. Athletes with EDs began both sport-specific training and dieting earlier. Onset of EDs was associated with prolonged dieting, weight fluctuation, sudden increase in training and traumatic events (e.g. injury)
Sundgot-Borgen and Torstveit [21]	Quantitative; cross-sectional, observational	EDs in athletes and non-athletes	1620 (960:660) [control group *N* = 1696]	Various; Norway	EDs were more likely to be observed in athletes relative to controls, with a higher prevalence observed in female athletes and leanness-dependent and weight-dependent sports. Management of EDs requires a collaborative effort (i.e. coaches, athletic trainers, parents, physicians and athletes)
Terry et al. [41]	Quantitative; cross-sectional, observational	Influences on eating, body shape perception, mood	103 (59:44)	Rowing; UK	Risk of EDs among elite rowers was moderated by age, sex and weight category; body shape concerns were greater for younger athletes, greater for heavyweights than lightweights and greater for females than for males. Results suggests that measures of mood may help identify athletes at risk of EDs
Terry and Waite [42]	Quantitative; cross-sectional, observational	Influence of age, sex and weight on EDs	124 (62:62)	Rowing; UK	Significantly higher eating attitude and body shape scores among lightweight athletes, with females’ body shape scores significantly higher than males’. Eating attitude and body shape were inversely correlated with age. Results suggested that the risk of EDs among elite rowers is mediated by age, sex and weight category
Torstveit et al. [43]	Quantitative; cross-sectional, observational	Disordered eating in female athletes	1276 (0:1276) [669 elite athletes, 607 controls] in part 1; 331 (0:331) [186 elite athletes, 145 controls] in part 2	Various; Norway	No differences between athletes and controls with respect to any of the criteria for disordered eating or clinical EDs. It was estimated that 28.1 and 20.8 % of the total population of athletes and controls, respectively, had clinical EDs. Predictors of clinical EDs were menstrual dysfunction in leanness athletes, self-reported EDs in non-leanness athletes and self-reported use of pathogenic weight-control methods in controls

*EDNOS* eating disorder not otherwise specified

**Table 4:** Summary of general-prevalence studies on mental health in elite athletes

Authors	Type	Purpose	*N* (male:female)	Sport; country	Main findings
Gouttebarge et al. [44]	Quantitative; cross-sectional, observational	Prevalence of mental health problems and psychosocial difficulties in current and former professional footballers	301 (301:0) [180 current players]	Football; Various	Prevalence of mental health problems ranged from 5 % (burnout) to 26 % (anxiety/depression) in current professional footballers. Prevalence of psychosocial difficulties ranged from 3 % (low self-esteem) to 26 % (adverse nutrition behaviour) in current professional footballers. Mental health problems were significantly associated with low social support and recent life events (former and current players). In current players, major life events in the previous 12 months were positively associated with distress, burnout and anxiety/depression. Low social support from trainer or coach was associated with burnout, and low social support from teammates was associated with anxiety/depression
Gulliver et al. [23]	Quantitative; cross-sectional, observational	Prevalence of mental health problems	224 (206:118)	Various; Australia	Overall, 46.4 % of athletes were experiencing symptoms of at least one of the mental health problems assessed, with rates consistent with findings in epidemiological studies of international athlete and community samples: depression (27.2 %), eating disorder (22.8 %), general psychological distress (16.5 %), social anxiety (14.7 %), generalised anxiety disorder (7.1 %) and panic disorder (4.5 %). Injured athletes had higher levels of both symptoms of depression and generalised anxiety disorder
Hammond et al. [45]	Quantitative; cross-sectional, observational	Prevalence of depression	50 (28:22)	Swimming; Canada	Before competition, 68 % of athletes met criteria for a major depressive episode within the previous 3 years (34 % current), with higher rates observed in female athletes. Prevalence halved after competition; however, 26 % self-reported mild to moderate symptoms of depression. Prevalence of depression doubled among the elite top 25 % of athletes assessed, and, within this group, performance failure was significantly associated with depression
Kotnik et al. [46]	Quantitative; cross-sectional, observational, comparison study	Psychological traits and any sex differences	62 (37:19)	Various; Slovakia	Female athletes reported greater anxiety, while male athletes reported higher self-confidence scores and masculinity. There were no sex differences in irritability, depression or neuroticism. There was a trend (*P* = 0.057) towards higher impulsivity scores in males. Stressful life situations tended to be managed by problem solving, logical analysis, positive reappraisal and seeking support
Mahoney [47]	Quantitative; cross-sectional, observational	Psychological variables associated with performance	67 (48:19)	Weight lifting; USA	Less interpersonal sensitivity, depression, psychoticism and psychological distress were reported in highly successful athletes. Trends were observed whereby successful athletes were more motivated (*P* < 0.07) and had higher self-esteem (*P* < 0.10). The most successful elite athletes indicated a composite picture of being less depressed and interpersonally sensitive yet more anxious and angry than their less successful counterparts
Meyers and Bourgeois [48]	Quantitative; cross-sectional, observational	Psychological skills of elite and ‘sub-elite’ equestrian athletes	54 (sex breakdown not reported)	Equestrian; USA	Elite competitors exhibited significantly higher scores for anxiety management and concentration than sub-elite athletes. Males exhibited greater vigour but less tension, depression, confusion and mood disturbance than females. Male athletes also scored higher in anxiety management and confidence. Female competitors indicated higher motivation
Morgan et al. [49]	Mixed method; cross-sectional, observational	Psychological characteristics and performance	14 (14:0)	Distance runners; USA	Athletes in this study showed low tension, depression, anger, fatigue and confusion scores, and high vigour scores. The measure of global mood and trait anxiety accounted for 45 % of the variance in athlete performance, highlighting the link between positive mental health and performance
Nixdorf et al. [50]	Quantitative; cross-sectional, observational	Prevalence of depressive symptoms and possible associated factors	134 (78:56) in elite group	Various; Germany	15 % prevalence of depression among elite athletes; higher levels of depressive symptoms observed among individual athletes than among team athletes. Depressive symptoms correlated with high levels of chronic stress, negative coping strategies and negative stress-recovery states. Results indicated that prevalence of depressive symptoms in elite athletes was comparable to that in the general German population
Schaal et al. [51]	Quantitative; cross-sectional, observational, retrospective	Psychological problems encountered, variations between sex and sport type	2067 (1339:728)	Various; France	17 % of athletes had at least one ongoing or recent mental health disorder; higher rates of psychopathology were observed in females. Female predominance applied to anxiety and eating disorders, depression, sleep problems and self-harming behaviours. Highest rates of generalised anxiety appeared in aesthetic sports. Eating disorders were most common among women in racing sports and men in combat sports
Wughalter and Gondola [52]	Quantitative; cross-sectional, observational	Psychological profile of elite athletes	16 (0:16)	Tennis; various	Older female athletes were significantly more likely to score higher on the vigour mood state and lower on all other mood states (tension, depression, anger, fatigue and confusion scores) than college-aged women

**Table 5:** Summary of substance use studies in elite athletes

Authors	Type	Purpose	*N* (male:female)	Sport; country	Main findings
Dietze et al. [54]	Quantitative; cross-sectional, observational	Alcohol consumption and alcohol-related harms	582 (582:0)	Australian Rules Football; Australia	In comparison with age- and sex-matched community scores, risky/high-risk consumption for long-term harm in players was lower during the playing season and higher during both the end-of-season period and the vacation period. Risky/high-risk drinking and short-term harm were frequent throughout the year, and reports of harmful effects of drinking and negative consequences were common (e.g. fighting while drinking). Club rules on alcohol consumption had little effect on outcome measures
Dunn et al. [55]	Quantitative; cross-sectional, observational	Prevalence of illicit drug use	974 (736:238)	Various; Australia	One third of the sample had opportunity to use illicit drugs in the previous year; overall prevalence was lower than that reported by the general population. 7 % of the sample indicated use of at least one illicit drug in the previous year, and one fifth reported having ever used cannabis. Knowing other athletes who used illicit drugs, being offered or having opportunity to use drugs and identifying as a ‘full-time athlete’ significantly predicted recent drug use
Dunn et al. [57]	Quantitative; cross-sectional, observational	Illicit drug use and consensus estimates	974 (736:238)	Various; Australia	Participants tended to report that there was a higher prevalence of drug use among athletes in general than among athletes in their sport, and these estimates appeared to be influenced by participants’ drug use history. While overestimation of drug use by participants was not common, this overestimation also appeared to be influenced by athletes’ drug use history
Dunn and Thomas [56]	Quantitative; cross-sectional, observational	Factors associated with illicit drug use	1684 (1212:472)	Various; Australia	8 % of the sample reported use of at least one illicit drug in the previous year. Predictors of use were identified: being offered or having opportunity to use illicit drugs in the previous year, knowing other athletes who used drugs and status as a full-time athlete. Athletes are part of a sports network (which includes family, coaches, support staff and other athletes), and these relationships may encourage use of, supply of and demand for drugs
Harcourt et al. [58]	Quantitative; longitudinal, experimental (no controls)	Illicit drug–testing programme	640 (640:0)	Australian Rules Football; Australia	Steady decline in the annual number of positive tests over the 7 years of the programme. An association between alcohol consumption and illicit drug use was observed. Illicit drug use was mostly conducted away from team mates. Using a harm minimisation strategy can work effectively alongside relevant anti-doping codes
O’Brien et al. [59]	Quantitative; cross-sectional, observational	Hazardous drinking and level of sport participation	430 (147:283) [270 in elite group]	Various; New Zealand	Elite sportspeople reported higher rates of hazardous drinking than non-sportspeople and non-elite sportspeople. International/country–level sportspeople also reported greater symptoms of dependence than other groups
O’Brien et al. [60]	Quantitative; cross-sectional, observational	Hazardous drinking and drinking motives	1214 (630:584) [275 in elite group]	Various; New Zealand	Elite provincial sportspeople reported the highest level of hazardous drinking, and elite international sportspeople reported the lowest. Elite provincial sportspeople and elite international sportspeople placed more emphasis on drinking as a way to cope with the stresses of participating in their sports
Thomas et al. [61]	Mixed method; cross-sectional, observational	Knowledge of illicit drugs and information seeking	974 (sex not reported)	Various; Australia	Athletes were confident in their knowledge of the effects of illicit drugs, such as cannabis and methamphetamine, but less confident in their knowledge of the effects of others (e.g. GHB and ketamine). Many felt that teammates would benefit from more information, delivered to athletes in a specific and relevant manner. Stigma was attached to information seeking
Waddington et al. [62]	Quantitative; cross-sectional, observational	Prevalence of illicit drug use	706 (sex not reported)	Football; England	Recreational drugs were commonly used by professional footballers; 45 % knew players who used recreational drugs. One third of players had not been tested for drugs within the preceding 2 years, and 60 % felt that they were unlikely to be tested in the next year

*GHB* gamma-hydroxybutyrate

**Table 6:** Summary of stress and coping studies in elite athletes

Authors	Type	Purpose	*N* (male:female)	Sport; country	Main findings
Anshel and Si [73]	Quantitative; cross-sectional, observational	Coping styles for acute stress	391 (253:138)	Various; China	Responses to stressful events were highly correlated with the athlete’s coping style (either approach or avoidance). Avoidance-coping style was more common than approach coping and was associated with turning one’s attention to the next task at hand, learning from the experience and perceiving the stressor as a normal part of the contest
Belem et al. [74]	Quantitative; cross-sectional, observational	Impact of coping strategies on resilience	48 (24:24)	Volleyball; Brazil	Athletes invited to the Brazilian team showed high levels of resilience. A number of coping skills impacted resilience: personal coping resources, coping with adversity, confidence and motivation, goal setting/mental preparation and coachability. Use of coping strategies to overcome problems, having defined goals, and motivation and concentration during competitions have a significant impact on development of a resilient profile in elite athletes
Devantier [75]	Quantitative; cross-sectional, observational	Psychological factors and injury vulnerability	87 (87:0)	Soccer; Denmark	Somatic anxiety and coping with adversity were the best predictors of injury severity. Coping with adversity was also a significant predictors of injury duration. Players with a history of previous injuries experienced more competitive trait anxiety than players not previously injured
Didymus and Fletcher [64]	Quantitative; longitudinal, observational	Coping strategies in response to organisational stressors	15 (8:7)	Swimming; UK	Employing one coping ‘family’ in isolation was perceived to be more effective than employing a combination of coping families. Self-reliance was perceived as the most effective coping family that was used in isolation, and escape and negotiation were perceived as the most effective combination of coping families. Stressful appraisals were associated with varied coping strategies
Dugdale et al. [76]	Quantitative; cross-sectional, observational	Coping with expected and unexpected stressors	91 (sex not reported)	Various; New Zealand	Unexpected stressors were perceived as more threatening than expected stressors. Athletes indicated a tendency to hold back from responding to unexpected stressors. Athletes used a variety of strategies to cope, with the highest ratings for acceptance, increasing efforts and planning. Venting of emotions, humour and denial were rated least frequently
Gastin [65]	Quantitative; longitudinal, observational	Monitored coping over the season	27 (sex not reported)	Australian Rules Football; Australia	Players generally coped well with the demands of elite competition; however, relative poor sleep quality was observed. Pain/stiffness and sleep quality had the highest average scores (poor). Subjective ratings of physical and psychological wellness were sensitive to changes to weekly training
Grove and Hanrahan [85]	Quantitative; cross-sectional, observational	Psychological strengths profile; athlete and coach comparison	39 athletes (15:24), 5 coaches	Field hockey; Australia	Ranking by players (greatest perceived strength to weakness): control of anxiety, maintaining concentration, planning and analysis, emotional control, use of imagery, maintaining self-confidence. Coaches perceived players to be relatively good at maintaining concentration and self-confidence, though relatively poor at controlling emotions and tension
Gutmann et al. [66]	Quantitative; longitudinal, observational	Psychological impact of training	11 (11:0)	Speed skating; USA	Stress reactivity and emotional lability likely to be detrimental to performance and characteristic of less experienced athletes. The most common acute stress reported was pain and fatigue, which had a cumulative effect on physical and psychological states. Associative and dissociative cognitive strategies were used to cope with acute stress. Setting daily goals, social support and maximising intrinsic rewards were used for coping with chronic stress
Ivarsson [67]	Quantitative; longitudinal, observational	Psychological predictors of injury	56 (38:18)	Soccer; Sweden	Trait anxiety, negative-life-event stress and daily hassles significantly predicted injury among professional soccer players, accounting for 24 % of variance
Johnson [79]	Quantitative; cross-sectional, observational, comparison study	Personality, mood and coping ability and injury	81 (65:16)	Various; Sweden	Injury was found to result in a depressed mood and in activation of coping strategies directed at receiving help. Female athletes become more anxious and tense, and used more emotion-focused coping strategies, than male athletes. Team-sport athletes were found to cope more in terms of ‘passive acceptance’ of help from others, whereas individual athletes were found to activate ‘problem-solving’ strategies in face of a stressor
Kristiansen et al. [77]	Mixed method; cross-sectional, observational	Relationship between task involvement and coping strategies	82 (60:22)	Wrestling; Europe	Being task involved was associated with use of more adaptive coping strategies (e.g. active coping, emotional support, instrumental support and positive reframing) than being ego involved. A total of 55 % of the variance in the choice of coping strategy was explained by task involvement (task orientation and mastery climate)
Kristiansen et al. [86]	Quantitative; cross-sectional, observational	Stress and motivation	82 (82:0)	Football; Europe	A mastery climate was directly and negatively associated with coach–athlete stress, while a performance climate was directly and positively associated with coach–athlete stress
Maestu et al. [68]	Quantitative; longitudinal, validation	Stress and recovery	12 (12:0)	Rowing; Estonia	Training volume associated with changes in fatigue (*R* = 0.66) and changes in general wellbeing (*R* = −0.62). Results demonstrated an increase in stress during a high-volume training period, and a decrease during the recovery period. An opposite effect was found in recovery scales
Mahoney and Avener [80]	Quantitative; cross-sectional, observational	Psychological factors and cognitive strategies	12 (12:0)	Gymnastics; USA	Self-verbalisations and certain forms of mental imagery differentiated Olympic-level and non-Olympic-level gymnasts. All finalists used imagery extensively, but the better athletes reported a higher frequency of ‘internal’ rather than ‘external’ images, and better gymnasts experienced greater self-confidence
Nicholls et al. [69]	Mixed method; longitudinal, observational	Stressors and coping strategies	8 (8:0)	Rugby; Europe	Frequently cited stressors were injury concerns, mental errors and physical errors, with a general decline in frequency as the season progressed. The most frequently cited coping strategies were increased concentration on task, blocking, positive reappraisal and increasing effort. Problem-focused coping strategies were used most frequently, followed by avoidance coping then emotion-focused coping
Nicholls et al. [71]	Mixed method; longitudinal, observational	Stressors and coping strategies	5 (5:0)	Rugby; various	Differences in stressors were identified in comparison of match and training days. Anxiety was the most cited emotion during training days and anger was the most cited emotion during match days. Coping effectiveness was greater during training than during matches. Emotional intensity was negatively associated with coping effectiveness
Nicholls et al. [70]	Mixed method; longitudinal, observational	Stressors and coping strategies	10 (5:5)	Cross-country running; UK	Stressors such as injury, illness and fatigue were more prominent during training than during competition. Athletes used more problem solving, planning, behaviour change and positive self-talk during training. Increasing effort and blocking were used more often during competition. Problem-focused coping strategies were associated with greater control of stressors, and a significant negative correlation occurred between stressor intensity and coping effectiveness
Noblet et al. [81]	Quantitative; cross-sectional, observational, validation	Stressors, job strain, psychological health	255 (255:0)	Australian Rules Football; Australia	Job control and work support were significant predictors of the dissatisfaction experienced by study participants. Only social support had a significant impact on both psychological health and job satisfaction outcomes. Strong links between club-based support and player wellbeing indicated that elite sporting organisations need to closely monitor the effectiveness of the social support provided by coaching staff, team mates and other club sources. The football-specific stressor that was predictive of both health and job satisfaction outcomes was post-football uncertainty
Pensgaard and Ursin [82]	Mixed method; cross-sectional, observational	Stressors and coping strategies	69 (49:20)	Various (winter sports); Norway	Stress was mainly experienced prior to competition. External distractions and expectations were the most frequently reported stress experiences. The coach was viewed as a major source of stress, with a subsequent lack of control and low satisfaction with performance. Type of stress was more detrimental to performance than time of experience
Pensgaard and Roberts [83]	Quantitative; cross-sectional, observational	Sources of distress, motivation, role of coach	69 (49:20)	Various (winter sports); Norway	Performance climate significantly predicted high total distress. Athletes with lower perceptions of ability perceived the coach to be more a source of distress than athletes with high perceptions. Perception of a mastery climate was negatively associated with the coach as a source of distress
Pensgaard and Roberts [84]	Mixed method; cross-sectional, observational	Sources of distress, motivation, role of coach	7 (5:2)	Skiing; Norway	All athletes rated very highly on task orientation, and in the moderate to high range on ego orientation. Most athletes perceived a high mastery climate and a low performance climate. Athletes emphasised the importance of the coach as creating the climate, as preferences for a supportive and caring climate
Wippert and Wippert [72]	Quantitative; longitudinal, observational	Stressors (career ending) and coping strategies	40 (17:23)	Skiing; Germany	Athletes who experienced supportive termination (involving discussion with coaches) acknowledged fewer symptoms of traumatic stress than those who experienced socially disintegrative termination. Nearly 20 % of participants acknowledged clinically relevant levels of traumatic stress at 3 and 8 months post-termination

**Table 7:** Summary of other mental health and wellbeing studies in elite athletes

Authors	Type	Purpose	*N* (male:female)	Sport, country	Main findings
Help seeking					
Gulliver et al. [11]	Quantitative; experimental, RCT	Internet-based intervention to increase mental health help seeking	59 (16:43)	Various; Australia	None of the interventions were efficacious in improving either attitudes, intentions or behaviour for mental health help seeking. Athletes had relatively high intention to seek help from formal sources at pre-intervention and more positive attitudes at pre-intervention than members of a general population sample of a similar age
Sleep					
Richmond et al. [53]	Quantitative; longitudinal, observational	Effects of interstate travel on sleep patterns	19 (19:0)	Australian Rules Football; Australia	In comparison with baseline, sleep duration was greater on the nights before home and away games (by 48 and 39 min, respectively, *P* < 0.05). Relative to home games, sleep ratings were poorer before away games (*P* < 0.05). Other sleep measures were unchanged. The authors concluded that interstate travel exerted minimal effect on sleep quality
Wellbeing					
Lundqvist and Raglin [63]	Quantitative; cross-sectional, observational	Psychological factors associated with wellbeing	104 (49:54)	Orienteers; Sweden	Level of psychological need dissatisfaction and perfectionistic concern, combined with self-esteem and need satisfaction, were all relevant indicators of elite athletes’ wellbeing and perceived stress profiles. Need dissatisfaction assessed independently from need satisfaction may act to influence the wellbeing/stress pattern

*RCT* randomised, controlled trial

#### Main Findings

Of the two included studies that focused on anger and aggression (see Table 1), one was conducted with rugby players [29], while the other was a case study of an elite table tennis player [30]. Anger tended to be experienced relatively frequently by the rugby players, was viewed as facilitative as opposed to debilitative and was positively associated with anxiety. In both studies, cognitive aspects (self-confidence or problem solving) were associated with less expression of anger in competition. No studies were found that evaluated off-field expressions of anger or aggression.

Four studies focused on anxiety in elite athletes (see Table 2): two on swimmers [32, 33] and two on athletes from mixed sporting populations [31, 34]. These studies focused primarily on the *performance* aspect of symptoms of anxiety (i.e. where athletic performance is evaluated as threatening and is associated with elevated levels of arousal or worry) as opposed to generalised clinical or subclinical experiences of non-competitive anxiety, which are summarised in the general-prevalence studies listed in Table 4. Athlete interpretation of anxiety states was identified as critical to the impact of anxiety. For example, a focus on performance (as opposed to cooperation and effort) predicted athlete worry [31], while interpretation of anxiety as facilitative was associated with more adaptive anxiety management strategies (i.e. approach-focused coping) [32] and performance levels [33]. Higher levels of athlete anxiety were also found to be related to negative patterns of perfectionism [34]. Recommendations to elite-level coaches included development of athlete skills in appraisal and interpretation of anxiety states [32] and the type of training culture facilitated among athletes (i.e. mastery as opposed to performance) [31].

Of the ten studies examining eating disorders and body image (see Table 3), six were conducted with mixed populations [21, 35–37, 40, 43], two with rowers [41, 42] and the remaining studies with distance runners [38] and figure skaters [39]. With the exception of the three studies that used an interviewer-administered diagnostic interview [35, 40, 43], all studies on eating disorders and body image used self-report data from standardised measures. Of the ten studies, five evaluated either eating disorder or body image issues in comparison with general community samples. The results from these studies were inconsistent. Three reported a higher incidence of eating disorder or body dissatisfaction in elite athletes relative to controls [36], especially in sports emphasising leanness or lower body weight [21, 35]. In contrast, one study found no difference between elite athletes and controls when the sample was restricted to females [43]. The included meta-analytic study that examined differences in body image reported no differences between athletes and non-athletes, or by sex or body mass index [37]; however, it must be noted this meta-analysis used homogenous inclusion criteria incorporating a large number of studies reporting data from non-elite athletes. The remaining studies identified athlete-specific risk factors for eating disorders or body image concerns, including sport-specific body-type demands (i.e. leanness) [38–40], age (higher risk in younger athletes) and sex (higher risk in females) [41, 42]. One study recruiting only males identified onset-related characteristics of eating disorders as commencement of training at an earlier age, dieting and experience of traumatic events, such as significant injury [40].

The ten general-prevalence studies on elite athlete mental health (see Table 4) reported data from either mixed samples [23, 46, 50, 51] or specific codes, including football [44], swimming [45], weight lifting [47], equestrian [48], distance running [49] and tennis [52]. Sample sizes for these studies varied on the basis of the population of interest, ranging from *N* = 2067 for a national-level study [51] to *N* = 14 for a study of distance runners. Of note, with the exception of one study utilising a structured diagnostic interview [45] and one study utilising clinician diagnoses [51], outcomes for general-prevalence studies were based on athlete self-report data from standardised measures. In the studies with larger sample sizes (*N* > 100), combined rates of high-prevalence disorders (i.e. mood or anxiety disorders) were frequently reported. In one large self-report study, up to 46.4 % of Australian athletes (*N* = 224; a mixed sample) met the clinical cut-off for a diagnosable mental health disorder based on standardised scales, with identified rates of depression (27.2 %), eating disorder (22.8 %) and anxiety disorder (social phobia; 14.7 %) [23]. Similar findings were reported for prevalence rates of depression (34 %) based on diagnostic interviews undertaken with swimmers [45] and self-reported depression and anxiety (26 %) in European football players [44], though rates of self-reported depression were lower (15 %) in a mixed sample of German elite athletes [50]. Markedly lower case rates, based on clinician diagnosis, were reported from a large French study (a mixed sample), with a lifetime prevalence rate for any disorder of 25.1 % and recent diagnosis rates of 8.6 % for anxiety, 4.9 % for eating disorder and 3.6 % for depression (and 0.6 % for suicidal ideation) [51]. This study did, however, report relatively frequent sleep problems (delayed onset, frequent waking and daytime drowsiness) in 21.5 % of athletes. Major life events, including injury and chronic stress, were associated with higher rates of distress, anxiety and depression [23, 44, 50]. The self-report general-prevalence studies reporting smaller sample sizes (*N* < 100) were relatively heterogeneous in outcomes. While male athletes tended to report lower anxiety than their female counterparts, sex differences in depression were inconsistent [46, 48]. Lower ratings of depression and distress were reported in highly achieving athletes [47] and in older versus younger female athletes [52], with global mood and anxiety predicting athlete performance [49].

The studies on elite athlete substance use focused on either alcohol [54, 59, 60], or other drugs [55–58, 61, 62] (see Table 5). Studies related to alcohol use indicated higher rates of consumption in athletes relative to the general community [59, 60]. However, during the playing season, the rates of risky alcohol use may be lower than general community levels [54]. Studies focusing on illicit drugs suggested relatively low use (7–8 %) in the previous year [55, 56] but significantly higher combined lifetime use when athletes were asked if they knew other athletes who used illicit drugs (up to 45 %) [62]. These studies reported that knowing another athlete who had used drugs was a significant predictor of their own use. In terms of drug-related attitudes, there was a tendency for athletes to overestimate levels of drug use in those competing in sports other than their own [56]. One intervention study that examined the long-term effects of an illicit drug–testing programme [58] found that rates of positive tests among athletes declined over an extended period, and the authors attributed this to education, harm minimisation and testing frequency. One study examined athlete knowledge regarding the effects of illicit drug use. Despite potential stigma relating to athletes seeking drug-related information (i.e. fellow players or staff assuming that information seeking equates to actual drug use), a substantial proportion of elite athletes expressed a desire to receive additional information regarding the effects of some classes of recreational drugs [61].

There were 22 studies examining stress and coping in elite athletes (see Table 6), comprising nine longitudinal studies [64–72] and 13 cross-sectional observational studies [73–85]. Injury, errors on the sporting field, fatigue and club/organisational climate were identified as common sources of stress among elite athletes [66, 68, 69, 71, 81, 86]. Four studies emphasised the impact of the coach in setting the organisational climate [72, 82–84], noting the negative implications of a performance culture over a mastery culture for athletes’ stress. Longitudinal studies found that stress related to injuries, external distractions and fatigue was highest during training periods [68–71], while opponents, officials/umpires and the crowd were the predominant sources of stress for athletes during competition [70].

The majority of the retrieved studies examined the different strategies employed by elite athletes to cope with the various stressors that were encountered [64–66, 70, 73–75, 79, 80, 85]. Adaptive, active coping strategies (such as problem solving, use of imagery, seeking social support and planning ahead) were frequently reported [66, 71, 81]; however, there was a tendency for athletes to engage in less adaptive (i.e. avoidance-coping) strategies when faced with unexpected stressors [73, 76]. One study [79] found that coping behaviour varied between sporting types, with athletes in team-based sports more likely to seek social support than individual competitors, and female athletes more likely to engage in strategies focused on managing affect. Coping strategies based on problem solving and behavioural change were found to be the most effective in managing stress [64, 70, 77] and developing resilience [74]. Emotional reactivity and stressful life events were associated with poor on-field performance and injury [66, 67, 75, 82].

There were single studies identified for the domains of help seeking [11], sleep [53] and wellbeing [63] (see Table 7). The help-seeking study was the only randomised, controlled trial that met the inclusion criteria. While this small study (*N* = 59) found no difference between athletes in the intervention conditions and control conditions with regard to attitudes, intentions and behaviours related to mental health help seeking, significant improvements were noted in depression and anxiety mental health literacy scores, as well as stigma, at 3-month follow-up [11]. The one study examining sleep reported on a small sample of Australian Rules footballers (*N* = 19) and found that match-related interstate air travel exerted relatively minimal effects on athlete sleep quality [53]. Finally, the study examining athlete wellbeing identified distinct profiles, with feeling unappreciated, greater perfectionism and lower self-esteem impacting on athlete general wellbeing [63].

## Discussion

Researchers have emphasised the limited peer-reviewed literature regarding the mental health and wellbeing of elite athletes [9, 19]. This narrative systematic review is the first to synthesise data from the existing knowledge base with the goal of identifying the incidence and/or nature of mental ill-health and substance use in *elite* athletes. Given the paucity of research in the field [19], the present review took a broad and inclusive approach to study both outcomes and designs. In doing so, it identified the relatively poor overall quality of study reporting to date and the lack of well-designed, intervention-based research in the area of elite athletes’ mental health and wellbeing. Despite the limitations of the extant literature, a number of key observations and tentative conclusions can be drawn from our data synthesis.

### Elite Athlete Vulnerability to Mental Illness

The data from studies reporting larger samples, although limited in scope, suggest that elite athletes experience a broadly comparable risk of high-prevalence mental disorders (i.e. anxiety, depression) relative to the general population [23]. That said, there may be subgroups of athletes at elevated risk of mental ill-health, including those in the retirement phase of their careers [44] or those experiencing performance failure [45]. As in the general population, major negative life events, including injury [23], may increase the risk of mental ill-health in elite athletes [50], though focused quantitative studies with adequate follow-up assessment periods are needed to confirm this. Findings regarding the prevalence of eating disorders and body image concerns relative to the general population were inconsistent. However, there was a tendency for higher vulnerability to these conditions in athletes involved in sports requiring a particularly lean body shape [21, 35, 38–40] and in female athletes [41–43]—the latter being consistent with the findings of general population studies [87]. Objective data, based on the results of medical review and tests, would likely assist in the assessment of eating pathologies and help counter the limitations of self-reporting (i.e. underreporting) [88]. Low social support was noted as a key risk factor for general mental ill-health, highlighting the importance of both formal and informal support networks for athletes [44, 46, 66]. All of the included general-prevalence studies were cross-sectional in nature. A natural advance for the field will be to assess athletes prospectively and better identify factors within the competitive spheres (i.e. performance or team success) and non-competitive spheres (i.e. approach coping, social support) for managing symptoms of mental ill-health. Given the significant overlap between the competitive years for elite athletes and the peak onset of mental disorders [10–12], future work should also assess low-prevalence disorders, such as psychosis or mania, in order to detect and direct at-risk athletes to early-intervention programmes or services.

### Elite Athlete Substance Use

Contextual factors also appear important for athlete substance use, though more rigorous studies are needed. For example, no research has examined illicit substance dependence in elite athletes. Nonetheless, higher rates of alcohol use may occur in elite athletes relative to the general population, though this may be largely due to a binge pattern of consumption during non-competitive or vacation periods [54]. Rates of self-reported illicit drug use were relatively low in the previous 12 months (i.e. 8 %) [56], which may be due to rigorous drug-testing procedures [58]. Further targeted research in the domain of athlete substance use is warranted, given the frequent harmful effects (e.g. fighting) reported [54] and the possibility of patterns of misuse developing in the transition to retirement. Given the possible stigma (i.e. assumed use) associated with elite athletes seeking drug information, improvements to specific, targeted and accessible (i.e. internet-based) information may be warranted [61].

### Athlete Coping

The literature related to athlete coping is more established than that for mental health outcomes. Most studies evaluated coping strategies employed by athletes to manage performance-related and non-performance-related stressors. In this way, coping was general in nature relative to psychosocial stressors (i.e. managing poor performance, injury or content on social media) rather than specific strategies for coping with a diagnosable disorder. Adaptive and maladaptive coping strategies were reported, though there was a lack of studies that sought to improve athlete coping. While a small-scale (*N* = 59), internet-based intervention failed to boost help-seeking-related attitudes, intentions or behaviours, increases were noted in mental health literacy [11]—an essential component of the help-seeking process. Given that stigma, poor mental health literacy and negative past experiences of mental health help seeking are key barriers for elite athletes [12], more well-designed studies, drawing on larger samples, are needed. Common athlete-specific stressors noted across studies included injury, poor performance, fatigue and organisational factors, such as the coaching environment and coaching expectations. The consistency of findings related to athlete stressors highlights these areas as potential avenues for targeted skills-based intervention programmes, including problem solving [70] and resilience training [74].

Management of athlete-specific stressors was also highlighted in the included studies focusing on athlete performance-related anxiety. Improved coping may be enabled by coaching staff emphasising a supportive training culture whereby athletes can interpret performance-related anxiety as facilitative, developing approach (as opposed to avoidance) strategies [31, 32]. Indeed, coaching staff themselves were identified as critical to setting the organisational climate—in turn, impacting on the level of stress experienced by athletes [72, 82–84]. Given the positive associations between coaches emphasising a mastery climate relative to a performance climate, future mental health intervention-based research should ensure the involvement and support of key coaching staff.

### Study Limitations and Future Directions

As indicated, the overall study quality in this field is poor, and heterogeneous study outcomes prevented the application of meta-analytic techniques. In addition, the nature of participant self-selection may have reduced the representativeness of the findings. While the included studies focused specifically on elite-level competition at the national or international level, differences between the included sports in terms of training, remuneration, media pressure and other salient variables must be considered [89]. Our study did not include athletes with disabilities—a population in which relatively little is known about mental health outcomes. Furthermore, most studies used self-reporting rather than a diagnostic interview [45]; therefore, the extent of psychiatric *disorders*, as opposed to mental health symptoms or probable ‘caseness’, in this population remains largely unknown. While some mental health domains were relatively well represented by the included studies, other domains—particularly athlete anger and aggression, help seeking and sleep—had very few studies. In addition, the included studies generally failed to include assessment of athlete psychological *strengths*, and only one included study assessed wellbeing-related outcomes. Further, there is a lack of research focusing on the transition from playing or competing in elite sport to retirement. Despite this, the current findings are useful for informing the next generation of studies focusing on elite athlete mental health.

The last two decades have witnessed extraordinary progress in sports medicine, performance coaching and elite athlete nutrition. As comparatively little progress has been made in the area of mental health, there is enormous scope for programmes to boost athlete wellbeing, which would likely flow on to benefits in competitive performance and increase the likelihood of a successful transition to retirement [24, 26, 49, 90]. Although vulnerability to mental ill-health might well be relatively comparable in elite athletes relative to the general community, there is significant scope for coaches, team psychologists and sport administrators to focus on targeted screening and early detection, monitoring and intervention—especially at key risk periods, such as significant injury [91, 92], transition to retirement [44, 93] and following performance difficulties [45]. Specific mental health help-seeking interventions are being developed for collegiate-level athletes, with a randomised, controlled trial currently underway [94]. The results of this work are likely to be relevant to elite-level athletes. Encouraging progress has been made in the development of mental health guidelines for working with school-aged athletes [95] and collegiate-level athletes [96–98]. These guidelines show a growing emphasis on the need to provide specific and targeted support for the mental health needs of athletes. They highlight the importance of monitoring changes in specific observable behaviours, appreciating psychological history and the need for a responsive crisis intervention framework specific to athletes. Development of comprehensive, targeted, disorder-specific treatment models are a required next step, and the National Athletic Trainers’ Association statement on preventing, detecting and managing disordered eating provides a useful disorder-specific model [99]. Psychoeducation should also extend to substance use—in particular, alcohol—given the tendency for hazardous use (bingeing) outside competition periods [100] and the stigma related to athlete help seeking in this domain [61].

Development of specific models of psychiatric intervention for elite athletes with significant psychopathology and impairment appears to be warranted [3, 101]. Such models should capitalise on an early-intervention framework [102–104], ensuring early detection and prompt access to high-quality, evidence-based interventions. This may include implementing mental health screening programmes alongside physical health checks [25], in addition to dissemination of mental health awareness support to key support people, including partners, friends, family, coaching staff and administrative staff. For this to occur, collaborative efforts would be required between sports medicine practitioners, psychiatrists, psychologists and other mental health professionals [105], mindful of overcoming treatment barriers and stigma for athletes at the elite level and balancing the need for treatment with the need for ongoing performance in, or commitment to, their chosen career [106].

In addition, from a broader public health perspective, better engagement of elite athletes in the domains of positive mental health (and as identifiable role models or ambassadors) may be significant in mental health stigma reduction and in boosting help-seeking behaviours and engagement in services. Importantly, the mental health of hard-to-engage populations, such as young men [107] and older men [108, 109], could be targeted. For this, the research will need to expand from simple cross-sectional designs and develop innovative strategies to improve athlete help seeking. Technology heralds a unique opportunity for this, especially given that the next generation of elite athletes will be digital natives and highly adept at utilising computer-based or internet-based interventions. Finally, given the paucity of high-quality studies reported to date, we encourage sporting bodies to consider public dissemination of any research (subject to ethical conduct) that is being conducted within the field of elite athlete mental health. Such efforts will enable the field to prosper and develop.

## Conclusion

Elite athletes experience a unique range of stressors that may potentially increase their vulnerability to mental ill-health. Key factors include the psychological impacts of injury, overtraining and burnout; intense public and media scrutiny; and managing ongoing competitive pressures to perform. For the assessment and management of the mental health needs of elite athletes’ to be on a par with their physical needs, more high-quality epidemiological and intervention studies are needed. Ideally, where possible and appropriate, the results of these should be disseminated beyond the organisation or sporting code.

## Electronic supplementary material

Below is the link to the electronic supplementary material.

Supplementary material 1 (DOCX 34 kb)
