# Supplementary material for: The Mental Health of Elite Athletes: A Narrative Systematic Review
Source: Sports Med. 2016 Feb 20;46(9):1333–53. doi: 10.1007/s40279-016-0492-2 (PMC4996886; doi:10.1007/s40279-016-0492-2)
Supplement: Supplementary file 1 — Supplementary material 1 (DOCX 34 kb) [file 40279_2016_492_MOESM1_ESM.docx]

**Electronic Supplementary Material Table S1: Database search strategy**

| **Database** | **Search terms** | **N (articles)** |
| --- | --- | --- |
| Embase | 1. 'athlete':ab,ti OR 'sport':ab,ti OR sport*:ab,ti 2. ('mental health' OR 'mental disorder' OR 'behavioral symptoms' OR 'resilience psychological' OR 'sleep' OR 'stress psychological' OR 'substance related disorders') 3..'wellbeing' OR 'coping' OR Support':.ab, ti 4.#1 AND #2 AND #3 | 438 |
| Pubmed | Search (((((athlete or sport[MeSH Terms])) OR sport*)) AND (((wellbeing or coping[MeSH Terms])) OR support[Other Term])) AND (mental health or mental disorders or behavioral symptoms or resilience, psychological, or sleep or stress, psychological or substance-related disorders[MeSH Terms]) | 665 |
| PsycInfo | S1.TI athlete OR AB athlete OR TI sport OR AB sport OR TI sport* OR AB sport* S2. TI Wellbeing OR AB Wellbeing OR TI Coping OR AB Coping OR TI Support OR AB Support S3. TX Mental disorder OR TX Mental health OR TX Behavioral symptom OR TX resilience, psychological OR TX sleep OR TX stress, psychological OR TX substance-related disorder S4. S1 AND S2 AND S3 | 396 |
| SportsDiscus | S1. TI ( athlete or sport or sport* ) OR AB ( athlete or sport or sport* ) AND TI ( wellbeing or coping or support ) OR AB ( wellbeing or coping or support ) S2.(TX ( mental health or mental disorder or behavioral symptom or resilience psychological, or stress, psychological or sleep or substance-related disorder )) AND (S5) | 620 |
| Cochrane | S1. "athlete" or "sport" or sport*:ti,ab,kw S2. "mental health'' "mental disorders" or "behavioral symptoms" or "resilience, psychological" or "sleep" or "stress, psychological" or "substance-related disorders" S3. "wellbeing" or "coping" or "support" ti,ab,kw s4. S1 AND S2 AND S3 | 79 |
| Google Scholar | Initial search from reference list of key papers retrieved in original search | 11 |
|  | Elite athlete mental health, elite athlete anxiety, elite athlete depression, elite athlete alcohol, elite athlete eating disorder | 28 |
|  | Elite athlete distress, elite athlete self-esteem, elite athlete quality of life, elite athlete personality, elite athlete psychotic OR psychosis, elite athlete drugs, elite athlete abuse, elite athlete substances, elite athlete sleep, elite athlete body image, elite athlete coping, elite athlete support, elite athlete mood | 25 |

**Electronic Supplementary Material Table S2: MeSH Headings and sub-headings used in search strategy**

| **Mesh headings** | **Sub headings** |
| --- | --- |
| Sports |  |
|  | Athletic Performance  Physical Endurance  Physical Fitness  Baseball  Basketball  Bicycling  Boxing  Football  Golf  Gymnastics  Hockey  Martial Arts  Tai Ji  Mountaineering  Racquet Sports  Tennis  Running  Jogging  Skating  Snow Sports  Skiing  Soccer  Sports for Persons with Disabilities  Swimming  Diving  Track and Field  Volleyball  Walking  Weight Lifting  Wrestling |
| Mental disorders | Adjustment Disorders  Anxiety Disorders  Agoraphobia  Neurocirculatory Asthenia  Obsessive-Compulsive Disorder +  Panic Disorder  Phobic Disorders  Stress Disorders, Traumatic +  Delirium, Dementia, Amnestic, Cognitive Disorders  Amnesia +  Cognition Disorders +  Consciousness Disorders  Delirium  Dementia +  Dyslexia, Acquired +  Dissociative Disorders  Multiple Personality Disorder  Eating Disorders  Anorexia Nervosa  Binge-Eating Disorder  Bulimia Nervosa  Female Athlete Triad Syndrome  Pica  Factitious Disorders  Munchausen Syndrome  Munchausen Syndrome by Proxy  Impulse Control Disorders  Firesetting Behavior  Gambling  Trichotillomania  Mental Disorders Diagnosed in Childhood  Anxiety, Separation  Attention Deficit and Disruptive Behavior Disorders +  Child Behavior Disorders  Child Development Disorders, Pervasive +  Communication Disorders +  Developmental Disabilities  Elimination Disorders +  Feeding and Eating Disorders of Childhood  Intellectual Disability  Learning Disorders +  Motor Skills Disorders  Mutism  Reactive Attachment Disorder  Schizophrenia, Childhood  Stereotypic Movement Disorder  Tic Disorders +  Mood Disorders  Affective Disorders, Psychotic +  Depressive Disorder +  Neurotic Disorders  Personality Disorders  Antisocial Personality Disorder  Borderline Personality Disorder  Compulsive Personality Disorder  Dependent Personality Disorder  Histrionic Personality Disorder +  Paranoid Personality Disorder  Passive-Aggressive Personality Disorder  Schizoid Personality Disorder  Schizotypal Personality Disorder  Schizophrenia and Disorders with Psychotic Features  Capgras Syndrome  Delusional Parasitosis  Morgellons Disease  Paranoid Disorders  Psychotic Disorders +  Schizophrenia +  Sexual and Sex Disorders  Disorders of Sex Development +  Sexual Dysfunctions, Psychological +  Sleep Disorders  Dyssomnias +  Parasomnias +  Somatoform Disorders  Body Dysmorphic Disorders  Conversion Disorder  Hypochondriasis  Neurasthenia  Substance-Related Disorders  Alcohol-Related Disorders +  Amphetamine-Related Disorders  Cocaine-Related Disorders  Inhalant Abuse  Marijuana Abuse  Neonatal Abstinence Syndrome  Opioid-Related Disorders +  Phencyclidine Abuse  Psychoses, Substance-Induced  Substance Abuse, Intravenous  Substance Withdrawal Syndrome +  Tobacco Use Disorder |
| Behavioural symptoms | Affective Symptoms  Aggression  Agonistic Behavior  Bullying  Catatonia  Child Reactive Disorders  Delusions  Depersonalization  Depression  Encopresis  Enuresis  Diurnal Enuresis  Nocturnal Enuresis  Hearing Loss, Functional  Human Coprophagia  Malingering  Mental Fatigue  Obsessive Behavior  Stalking  Paranoid Behavior  Polydipsia, Psychogenic  Schizophrenic Language  Self-Injurious Behavior  Self Mutilation  Suicide +  Stress, Psychological  Wandering Behavior |
| Substance-related disorders | Alcohol-Related Disorders  Alcohol Amnestic Disorder +  Alcohol Withdrawal Delirium  Alcoholic Intoxication  Alcoholism  Binge Drinking  Psychoses, Alcoholic  Wernicke Encephalopathy  Amphetamine-Related Disorders  Cocaine-Related Disorders  Inhalant Abuse  Marijuana Abuse  Neonatal Abstinence Syndrome  Opioid-Related Disorders  Heroin Dependence  Morphine Dependence  Phencyclidine Abuse  Psychoses, Substance-Induced  Substance Abuse, Intravenous  Substance Withdrawal Syndrome  Alcohol Withdrawal Delirium  Tobacco Use Disorder |
| Sleep | Dreams  Sleep Deprivation  Sleep Stages  Sleep, REM |
| Stress, psychological | Burnout, Professional |
| Psychological phenomena and processes | Mental Health  Sleep +  Stress, Psychological +  Resilience, Psychological |

**Electronic Supplementary Material Table S33: Methodological quality assessment of included studies**

| **Author(s)** | **Study participants well defined (time, place, personal characteristics)** | **Selection random (1) or consecutive (0)** | **Participant rate >80% OR if participant rate is low, comparison respondents/non-respondents described** | **Standardised, validated questionnaire OR clear description of outcomes measured** | **Disclosure of ethical review** | **Number of criteria met (out of 5)** |
| --- | --- | --- | --- | --- | --- | --- |
| Abrahamsen et al. (2008) | 1 | 0 | 0 | 1 | 1 | **3** |
| Anshel & Si (2008) | 1 | 0 | 1 | 1 | 0 | **3** |
| Belem et al. (2014) | 1 | 0 | 1 | 1 | 1 | **4** |
| Byrne & McLean (2002) | 1 | 0 | 1 | 1 | 0 | **3** |
| Devantier (2011) | 1 | 0 | 0 | 1 | 0 | **2** |
| Didymus & Fletcher (2014) | 1 | 0 | 1 | 1 | 1 | **4** |
| Dietze et al. (2008) | 1 | 0 | 1 | 1 | 1 | **4** |
| Dugdale et al. (2002) | 1 | 0 | 0 | 1 | 0 | **2** |
| Dunn et al. (2011) | 1 | 0 | 1 | 1 | 1 | **4** |
| Dunn et al. (2012) | 1 | 0 | 1 | 1 | 0 | **3** |
| Dunn & Thomas (2012) | 1 | 0 | 1 | 0 | 1 | **3** |
| Filaire et al. (2007) | 1 | 0 | 0 | 1 | 1 | **3** |
| Gastin (2013) | 1 | 0 | 1 | 1 | 1 | **4** |
| Gouttebarge et al. (2015) | 1 | 1 | 0 | 1 | 1 | **4** |
| Grove & Hanrahan (1988) | 1 | 0 | Not reported | 1 | 0 | **2** |
| Gutmann et al. (1984) | 1 | 0 | 1 | 1 | 0 | **3** |
| Gulliver et al. (2012) | 1 | 0 | 0 | 1 | 1 | **3** |
| Gulliver et al. (2015) | 1 | 0 | 0 | 1 | 1 | **3** |
| Hammond et al. (2013) | 1 | 0 | Not reported | 1 | 1 | **3** |
| Harcourt et al. (2012) | 1 | 1 | 1 | 1 | 1 | **5** |
| Hatzigeorgiadis & Chroni (2007) | 1 | 0 | Not reported | 1 | 0 | **2** |
| Hausenblas & Symons Downs (2001) | 1 | N/A | N/A | 1 | N/A | **2** |
| Hulley & Hill (2001) | 1 | 0 | 1 | 1 | 0 | **3** |
| Ivarsson (2013) | 1 | 0 | Not reported | 1 | 1 | **3** |
| Johnson (1997) | 1 | 0 | Not reported | 1 | 0 | **2** |
| Jones et al. (1994) | 1 | 0 | Not reported | 1 | 0 | **2** |
| Jonnalagadda et al. (2004) | 1 | 0 | Not reported | 1 | 1 | **3** |
| Koivula et al. (2002) | 1 | 0 | Not reported | 1 | 0 | **2** |
| Kotnik et al. (2012) | 1 | 0 | 1 | 1 | 1 | **4** |
| Kristiansen et al. (2008) | 1 | 0 | Not reported | 1 | 0 | **2** |
| Kristiansen et al. (2012) | 1 | 0 | 1 | 1 | 1 | **4** |
| Lundqvist & Raglin (2014) | 1 | 0 | 0 | 1 | 1 | **3** |
| Maestu et al. (2003) | 1 | 0 | 1 | 1 | 1 | **4** |
| Mahoney (1989) | 1 | 0 | 0 | 1 | 0 | **2** |
| Mahoney & Avener (1977) | 0 | 0 | 1 | 1 | 0 | **2** |
| Meyers & Bourgeois (1999) | 1 | 1 | 1 | 1 | 1 | **5** |
| Morgan et al. (1988) | 1 | 0 | Not reported | 1 | 0 | **2** |
| Nicholls et al. (2006) | 1 | 0 | Not reported | 1 | 0 | **2** |
| Nicholls et al. (2009) | 1 | 0 | Not reported | 1 | 1 | **3** |
| Nicholls et al. (2009) | 1 | 0 | Not reported | 1 | 0 | **2** |
| Nixdorf et al. (2013) | 1 | 0 | 0 | 1 | 0 | **2** |
| Noblet et al. (2003) | 1 | 0 | 1 | 1 | 0 | **3** |
| O'Brien et al. (2005) | 1 | 0 | 1 | 1 | 0 | **3** |
| O'Brien et al. (2007) | 1 | 0 | 1 | 1 | 1 | **4** |
| Pensgaard & Ursin (1998) | 1 | 0 | 0 | 1 | 0 | **2** |
| Pensgaard & Roberts (2000) | 1 | 0 | 0 | 1 | 0 | **2** |
| Pensgaard & Roberts (2002) | 1 | 0 | 0 | 1 | 0 | **2** |
| Richmond et al. (2007) | 1 | 0 | 1 | 1 | 1 | **4** |
| Robazza & Bortoli (2007) | 1 | 0 | Not reported | 1 | 0 | **2** |
| Schaal et al. (2011) | 1 | 0 | N/A | 1 | 1 | **3** |
| Si & Lee (2008) | 1 | 0 | N/A | 1 | 0 | **2** |
| Sundgot-Borgen (1994) | 1 | 0 | 1 | 1 | 0 | **3** |
| Sundgot-Borgen & Torstveit (2004) | 1 | 0 | 0 | 1 | 1 | **3** |
| Terry et al. (1999) | 1 | 0 | Not reported | 1 | 0 | **2** |
| Terry & Waite (1996) | 1 | 0 | 1 | 1 | 0 | **3** |
| Thomas et al. (2011) | 1 | 0 | 1 | 1 | 1 | **4** |
| Torstveit et al. (2008) | 1 | 0 | 1 | 1 | 1 | **4** |
| Waddington et al. (2005) | 1 | 0 | 0 | 1 | 0 | **2** |
| Wippert & Wippert (2008) | 1 | 0 | 0 | 1 | 0 | **2** |
| Wughalter & Gondola (1991) | 1 | 0 | Not reported | 1 | 0 | **2** |
| **Total** | **59** | **3** | **25** | **59** | **27** | **M=2.88** |
